# Plants’ Response to Abiotic Stress: Mechanisms and Strategies

**DOI:** 10.3390/ijms241310915

**Published:** 2023-06-30

**Authors:** Yan Zhang, Jing Xu, Ruofan Li, Yanrui Ge, Yufei Li, Ruili Li

**Affiliations:** 1 State Key Laboratory of Tree Genetics and Breeding, College of Biological Sciences and Technology, Beijing Forestry University, Beijing 100083, China; zhangyan_yolo@bjfu.edu.cn (Y.Z.); xujing@bjfu.edu.cn (J.X.); liruofan@bjfu.edu.cn (R.L.); geyanrui@bjfu.edu.cn (Y.G.); liyufei0371@outlook.com (Y.L.); 2 National Engineering Research Center of Tree Breeding and Ecological Restoration, College of Biological Sciences and Technology, Beijing Forestry University, Beijing 100083, China; 3 The Tree and Ornamental Plant Breeding and Biotechnology Laboratory of National Forestry and Grassland Administration, College of Biological Sciences and Technology, Beijing Forestry University, Beijing 100083, China; 4 Institute of Tree Development and Genome Editing, Beijing Forestry University, Beijing 100083, China

**Keywords:** abiotic stress, stress sensors, signal transduction, response

## Abstract

Abiotic stress is the adverse effect of any abiotic factor on a plant in a given environment, impacting plants’ growth and development. These stress factors, such as drought, salinity, and extreme temperatures, are often interrelated or in conjunction with each other. Plants have evolved mechanisms to sense these environmental challenges and make adjustments to their growth in order to survive and reproduce. In this review, we summarized recent studies on plant stress sensing and its regulatory mechanism, emphasizing signal transduction and regulation at multiple levels. Then we presented several strategies to improve plant growth under stress based on current progress. Finally, we discussed the implications of research on plant response to abiotic stresses for high-yielding crops and agricultural sustainability. Studying stress signaling and regulation is critical to understand abiotic stress responses in plants to generate stress-resistant crops and improve agricultural sustainability.

## 1. Introduction

**Figure 1:**
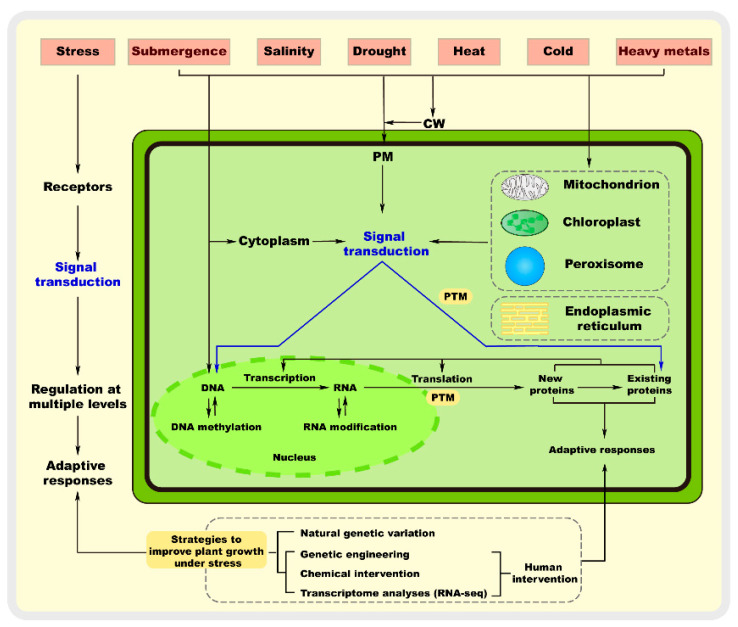
Plant resistance to abiotic stresses at the molecular level through sensing mechanisms and genetic responses. Abiotic stress can be perceived in different cellular compartments, including the cell wall (CW), plasma membrane (PM), cytoplasm, mitochondria, chloroplasts, peroxisomes, endoplasmic reticulum, and nucleus, leading to the initiation of molecular responses. These stress sensors then transmit the signals downstream through secondary messengers and regulatory proteins, such as Ca^2+^, ROS, and protein kinases. Furthermore, it is now well known that abiotic stress induces different responses involving stress sensing, signal transduction, and regulation at multiple levels. Therefore, plants have evolved mechanisms to adjust their growth to survive and reproduce under stress. PTM: post-translational modifications.

For immobile plants, abiotic environmental factors are often the main detrimental factors affecting their growth and development [1]. Abiotic stress refers to the adverse effect of any abiotic factor on a plant in a given environment, resulting in a range of responses, from changes affecting biological processes such as gene expression and cell metabolism to growth and development [2]. Specifically, abiotic stress includes extreme temperature stress, drought stress, flooding stress, salinity stress, metal stress, and nutrient stress, and different stresses can cause different responses [3]. Extreme temperatures, drought, and saline soils are the main environmental factors that limit the survival and interrelated distribution of plants in nature [1]. For example, high temperatures and drought often occur together. The perception and transduction of and response of plants to stress signals are of great scientific interest as an important biological issue [4]. In particular, the study of transduction signals at all levels has provided strong evidence for various stress responses in plants [5]. Both salt and drought stress cause osmotic stress, which involves the regulation of a wide range of inorganic and organic metabolites and results in damage to plants, including ion toxicity, reactive oxygen species (ROS) accumulation, plasma membrane disruption, and cell wall damage [6]. Non-adaptive changes are caused by protein misfolding and the disruption of the cell wall structure in response to stress in plants, whereas adaptive changes lead to increased plant resistance [7]. Thus, the molecular mechanism of plant response to abiotic stress is multi-level and multi-process, involving sensing, signal transduction, transcription, processing, and protein translation and modification, and is a complex response mechanism with multiple genes, signaling pathways, and metabolic processes (Figure 1) [8].

As the world’s population continues to grow, food security is becoming a major issue that is further complicated by the potential impact of climate change on crop productivity [1]. Extreme temperatures, drought, and soil salinization are the main adverse environmental conditions affecting crops [3]. The impact of abiotic stresses on plant breeding and production is therefore also of great interest. Using genetic engineering, scientists have attempted to reduce the negative effects of stress on agricultural production but with limited success [2]. Although a lot of genes involved in plant abiotic stress signaling and response have been identified [7], it remains a challenge to apply this knowledge to crop production with increased stress resistance. Energy and resource constraints are typically used to explain the seemingly inevitable trade-off between growth and stress resistance: under stress, plants must divert energy and resources away from growth and toward a stress response [4]. However, increasing evidence suggests that under stress conditions, plants actively repress growth as an adaptive strategy to maximize survival [1].

The stress-response program in plants is sensitive to mild stresses, preparing the plants for the possibility of more severe stress in the future. Early in the stress response, the stress signaling network actively suppresses cellular anabolic activities and plant growth even when the cellular energy status is unaffected [6]. This review summarizes recent research on the categories and molecular mechanisms of abiotic stress in plants, including signal transduction and expression regulation, and provides an outlook on future research directions. It may be necessary to understand the reciprocal regulation between the genetic programs for stress response and growth and thereby engineer hardier high-yielding crops.

## 2. Stress Sensing and Regulatory Mechanism

### 2.1. Stress Sensing

#### 2.1.1. Changes in Osmolarity

Abiotic stresses such as high salinity and drought can produce direct or indirect hyperosmotic stress in plants [9]. The adaptation of plant cells to stressful environments by increasing the solute concentration, mainly through the accumulation of various inorganic and organic substances, thereby reducing the osmotic potential and enhancing the plant’s ability to retain water, is known as osmoregulation [10]. The substances involved in osmoregulation are broadly divided into two categories, namely, inorganic ions, such as K^+^, Na^+^, and Ca^2+^, and organic solutes, such as proline, betaine, soluble sugars (SSs), and polyols [9,11]. During drought, naked fruitwood leaves can reduce their osmotic potential through accumulating Pro, SS, and soluble protein (SP), thus providing a degree of drought resistance [12]. As soil drought gradually increases, the proline content accumulates significantly, but the increasing rate declines; the SP content first decreases and then starts to increase significantly when the relative soil water content drops below 31.38%; and the SS content continues to increase [13]. In rice (Oryza sativa), hyperosmotic conditions lead to the opening of OSCA ion channels and Ca^2+^ translocation into the cells [14,15]. Osmotic stress treatment also rapidly activates the SnRK2 protein kinase family. Research data have suggested that SnRK2s are required for plant tolerance to osmotic stress [16]. Osmotic stress leads to the production of various lipid signals, such as sphingolipids and phosphatidic acid [17]. Stress also induces the production of some adversity proteins in plant cells, mainly osmoregulatory proteins, the production of which is beneficial in reducing the osmotic potential of the cells and preventing cell dehydration, which helps to improve the resistance of plants to osmotic stress.

In addition, autophagy is widely involved in plant growth, development, and stress response and plays an important role in plant resistance responses. The autophagy mechanism regulated by *ATG* genes is a conserved degradation pathway [18]. The role of *ATG* genes as an important gene family for stress resistance has now been demonstrated in a variety of plants [19,20,21,22,23].

#### 2.1.2. Changes in Salinity

Soil salinity is an important factor that affects agricultural productivity and ecology globally. High soil salinity severely affects plant growth and development, and excess salt ions cause damage to plants mainly through triggering osmotic stress, ion toxicity, and oxidative stress. In turn, plants respond by regulating stomatal opening, synthesizing osmolytes, compartmentalizing excess ions, and scavenging ROS to reduce stress damage [24]. The osmotic stress signal generated early in salt stress causes plants to be more tolerant of high salt and drought through reducing stomatal opening to reduce water loss [25]. Moreover, this signal accelerates the rate of stomatal movement under dynamic light, and osmotic stress accelerates stomatal closure when switching between strong and weak light intensities [26]. Plants are able to efficiently use Na^+^ and Cl^−^ in vesicles and organic solutes in vivo for osmoregulation to avoid ion toxicity. However, plants grown at high salinity require altered cell wall structure or water–salt movement pathways for energy-efficient osmoregulation [27]. AtCBL4 (also known as AtSOS3) in the *Arabidopsis* salt overly sensitive (SOS) signaling pathway acts mainly in the roots, where it activates the protein kinase AtCIPK24 (also known as AtSOS2) through receiving Ca^2+^ signals. Activated AtSOS2 not only phosphorylates the Na^+^/H^+^ reverse transporter AtSOS1 located at the plasma membrane [28] but may also phosphorylate the Na^+^/H^+^ reverse transporter AtNHX on the vesicle membrane, thus enabling root cells to expel Na^+^ from the cytoplasm through AtSOS1 and compartmentalize Na^+^ from the cytoplasm to the vesicle through AtNHX, thereby reducing the toxic effect of Na^+^ in the cytoplasm and maintaining a relatively stable K^+^/Na^+^ ratio in the cytoplasm [29]. In addition, the transport of Na^+^ into the vesicles can increase the osmotic pressure of the vesicles, which allows the cells to absorb water from outside to reduce osmotic stress. High salinity also triggers changes in lipid membranes and disrupts cell wall organization through a variety of pathways, including the replacement of pectin-crosslinked Ca^2+^ and the accumulation of ROS through the cross-linking of phenols and cell wall glycoproteins leading to cell wall hardening [30]. Under abiotic stress, the B-box (BBX) family transcription factor lbBBX24 activates the expression of the peroxidase gene lbPRX17, and the lbBBX24-lbTOE3-lbPRX17 module improves salt tolerance in sweet potato plants through scavenging ROS in sweet potato plants [31]. Studying the response mechanisms and response modules of plants in response to abiotic stress is important for selecting superior varieties with strong abiotic stress tolerance.

#### 2.1.3. Changes in Temperature

Cold stress rapidly induces the expression of many transcription factors, including the AP2 structural domain protein CBF, which then activates the expression of many downstream cold response (COR) genes [32]. Membrane fluidity and the cytoskeleton, as well as inward calcium flow, are involved in the regulation of cold stress by COR genes and mitogen-activated protein kinase (MAPK) [33]. Several cold stress sensors have been proposed that are based on plasma membrane-localized Ca^2+^ channels or Ca^2+^ channel regulators [34,35,36,37]. In *Arabidopsis*, mutations in AtANN1 result in the loss of function, significantly affecting Ca^2+^ influx and reducing freezing resistance [35]. The accumulation of CBF transcription factors can increase photoreceptor (phyB) stability to promote freezing resistance in plants [38].

Heat stress leads to protein denaturation and therefore requires the expression of heat shock proteins (HSPs), many of which act as molecular chaperones to prevent protein denaturation and maintain proteostasis [39]. HSPs are usually found in the cytoplasm, endoplasmic reticulum, mitochondria, and other parts of plants [40]. They can be divided into five families (HSP70, sHSP, HSP100, HSP60, and HSP90) according to their molecular weight [41]. Among them, HSP70 is one of the most abundant heat stress proteins in eukaryotic cells, and its expression level is influenced by the external environment. These different families of HSPs have been identified and studied in different plants [42,43,44,45,46,47,48]. In previous research, *Arabidopsis* seedlings showed a rapid increase in *HSP70* family gene expression under dramatic changes in growth temperature [40,49]. Under high-temperature stress at 40 °C, rice *HSP70* genes were significantly expressed within a very short period of time [50]. At high temperatures, misfolded proteins accumulate and bind to HSPs, which release heat stress transcription factors (HSFs) to activate the heat stress response. Heat stress also activates MAPK, which regulates *HSP* gene expression, and MAPK activation may be associated with heat-induced changes in membrane fluidity and calcium signaling, which are important for *HSP* gene expression and heat resistance [33]. Common features between cold and heat stress signaling are not only limited to changes in membrane fluidity, calcium signaling, and MAPK activation but also include the involvement of phospholipid signaling, NO, proteasomal degradation, and ROS [32,39].

### 2.2. Signal Transduction

Stress-specific signal transduction is triggered by the perception of adverse environmental conditions [30]. This process involves various second messengers, such as Ca^2+^, ROS, nitric oxide (NO), and phospholipids, as well as different types of protein kinases. After the stress is sensed by the plant cell, signals are transmitted and amplified by these second messengers [1].

#### 2.2.1. Ca^2+^ Signaling

Different stresses, such as cold, drought, and high salinity, have certain common features in terms of their effects on plants and the ways that plants perceive them. For example, these abiotic stresses usually cause osmotic stress in plant cells [1]. In addition, they also rapidly induce a transient increase in intracellular Ca^2+^ concentrations [8]. Thus, Ca^2+^ is considered to be a universal second messenger in primary stress signals due to its properties that make it suitable for functioning as a ubiquitous signaling molecule [8]. In comparison with other internal or external spaces, the low concentration of Ca^2+^ in the cytoplasm makes the concentration easily changeable. A number of proteins can recognize and interrupt [Ca^2+^] changes [51]. Moreover, there are many Ca^2+^ osmotic channels or transporters that can precisely control these concentration changes. In addition, stress-induced increases in cytoplasmic calcium concentration have been found to vary in intensity, frequency, and subcellular location [30].

Transient Ca^2+^ can be detected in *Arabidopsis* guard cells within 15 s after osmotic stress treatment [8]. Calcium signals can then be detected by calcium-binding proteins, which usually feed the signal to an interacting protein kinase or to a kinase directly fused to them, such as the calcium-dependent protein kinases (CDPKs or CPKs) [52]. During salt stress, a specific cytosolic Ca^2+^ signal is perceived by the SOS pathway in *Arabidopsis* thaliana [53]. In the SOS pathway, SOS3 or SCaBP8 interacts with a protein of the SnRK3 kinase family (also known as CIPK) and activates SOS2. Many combinations of SCaBP/CBL-CIPK complexes are present in plants [54]. Similar to the SOS3-SOS2 module, these complexes play an important role in Ca^2+^-mediated responses to various abiotic stresses, especially those involving the regulation of ion transport protein activity [1].

#### 2.2.2. ROS Signaling

The production of ROS, including superoxide anion, H_2_O_2_, hydroxyl radical, and singlet oxygen, is a major feature of plant responses to various abiotic stresses [1]. In plants, ROS can be produced in multiple organelles, including chloroplasts, mitochondria, and peroxisomes, or by the plasma membrane-localized Rboh NADPH oxidases. In particular, apoplastic ROS produced by respiratory burst oxidase homologs D and F (RbohD and RbohF, respectively) may stimulate specific calcium and electrical signals and mediate rapid systemic signaling in response to stress [55]. Within *Arabidopsis*, this type of signal was found to propagate at ~8.4 cm per minute. Although ROS are detrimental to biomolecules when their levels exceed the cellular capacity for detoxification, they also play important parts in stress signaling, including high light stress-induced retrograde signaling (which starts in the chloroplast and induces stress responses in the nucleus) and abscisic acid (ABA) signaling [1].

#### 2.2.3. Protein Phosphorylation

Protein phosphorylation is a widespread and critical event in signal transduction that occurs in plants in response to different abiotic stress conditions. Members of the protein phosphatase type 2C (PP2C) family and the SnRK2 subfamily of protein kinases are key players in various stress signaling pathways, and these remain unchanged in crops such as rice and maize [56]. These protein kinases regulate various downstream proteins, including transcription factors; the plasma membrane anion channel SLAC1, which controls stomatal closure; and the plasma membrane NADPH oxidase RbohF, which produces extracellular hydrogen peroxide (H_2_O_2_) [57,58]. In addition to the SnRKs, the RLKs and the kinase cascade consisting of MAPK, MAP kinase (MAP2K), and MAP3K are frequently involved in stress signaling [59]. During stress responses, the MAPK cascade may or may not be involved in calcium signaling. For example, in *Arabidopsis*, the MEKK1-MKK2-MPK4 cascade is involved in cold-induced Ca^2+^ signaling via the calcium/calcium-regulated receptor-like kinase CRLK1, whereas the MAP3K17/18MKK3-MPK1/2/7/14 cascade acts downstream of SnRK2s in response to ABA and is therefore not significantly involved in Ca^2+^ signaling [60,61].

#### 2.2.4. Abscisic Acid (ABA) Signaling

ABA plays an important role in a variety of responses to abiotic stresses and is often considered a stress hormone [1]. ABA is an isoprenoid hormone that is synthesized from carotenoids. ABA mediates developmental processes such as seed maturation and dormancy and stress responses, including stomatal closure, leaf senescence, and growth inhibition [3]. ABA signaling pathways are critical for plant responses to drought and salt stresses. One of the most important advances in stress signaling in the last decade has been the identification of ABA receptors and the elucidation of key ABA signaling pathways. RCAR (ABA receptor regulatory component), PYR1 (pyrazine resistance 1), PYL (PYR1-like) protein, referred to as “PYL,” and protein phosphatase type 2C (PP2C) are ABA receptors [56,62]. When stress signals stimulate ABA synthesis, ABA enters the hydrophobic binding pocket of the PYL START structural domain, inducing a conformational change that closes the pocket and provides a surface for PP2C binding [63]. When ABA binds to PYL and PP2C, SnRK2s release the inhibitory effects of PP2Cs and are then able to phosphorylate downstream substrates, such as ABF/AREB (ABA response element-binding factor/ABA response element-binding protein) transcription factors, which regulate genetic responses to stress; Rbohs, which produce ROS; and ion channels that close stomata [64,65].

ABA signaling is initiated by ABA receptor binding, which causes the ABA receptor to interact with and inhibit the activity of PP2C, which in turn leads to the activation of SnRKs or other kinases [3]. These activated protein kinases regulate the activity of transcription factors to control the expression of stress-responsive genes. The PYL-PP2C-SnRK2 module is regulated by a variety of proteins [3].

### 2.3. Regulation Mechanism of Abiotic Stress in Plants

#### 2.3.1. Transcriptional Regulation

In addition to protecting plants from abiotic stresses and thus re-establishing ion and water homeostasis through inducing rapid regulation, stress-induced signaling also induces genome-wide transgenomic reprogramming, which activates additional protective mechanisms such as osmoregulation, detoxification, and the repair of stress-induced damage [1]. Currently, many transcription factors (TFs) in different plants have been identified via genome-wide analysis, such as NF-Y, WOX, WRKY, bZIP, and NAC [6,66,67,68,69]. Certain key transcription factors involved in plant responses to abiotic stress are summarized in Table 1. Stress-specific transcriptional patterns are linked to upstream signaling through transcription factors. In addition to stress-specific patterns, many different stresses, such as cold stress, hypertonic stress, and hypersalinity stress, can also induce common transcriptional responses [70]. In general, these common stress response genes encode proteins associated with MAPK cascades, calcium signaling, ROS, apoptosis, and protein degradation and are located in various cellular compartments [70].

As discussed above, abiotic stresses such as drought and high salinity induce the biosynthesis of ABA, which then mediates stress response through phosphorylation-dependent signaling cascades [1]. Although hundreds of genes are transcriptionally regulated by drought, high salt, and cold stresses through ABA signaling, many stress-responsive genes are independently activated by ABA pathways. The ABA-responsive cis-element (ABRE) ACGTGG/TC was found in the promoter areas of several ABA-regulated genes, whereas the dehydration-responsive cis-element (DRE) TACCGACAT was found in the promoter regions of ABA-independent cold-responsive and drought-responsive genes [71]. The transcription factors CBF (also known as DREB1) and DREB2 bind to the DRE and activate the expression of their target genes. The *CBF4* and *DREB2* genes were primarily induced by osmotic stress or high salinity, while *CBF1/2/3* genes are specifically induced by cold, thus contributing to the stress-specific regulation of downstream genes, and many stress-responsive genes are induced by ABA mechanisms independently [72].

**Table 1:** Key transcription factors involved in plant responses to abiotic stress.

Transcriptional Factor	Plant Species	Stress	References
*NF-Ys*	*Populus trichocarpa* *Glycine max* *Arabidopsis thaliana* *Solanum tuberosum* *Zea mays*	Drought Salt Nutrient Osmotic	[2,73,74,75,76,77]
*WOXs*	*Oryza sativa* *Populus nigra* *Arabidopsis thaliana* *Gossypium hirsutum*	Drought Cold Salt	[78,79,80,81,82,83,84]
*WRKYs*	*Malus x domes* *Sorghum bicolor* *Fortunella crasifolia* *Zea mays* *Pyrus betulaefolia*	Salt Temperature Drought	[10,85,86,87,88,89]
*MYB*	*Arabidopsis thaliana* *Zea mays* *Astragalus membranaceus*	Salt Cold Drought	[66,90,91]
*bZIP*	*Apium graveolens* *Ipomoea batatas* *Triticum aestivum* *Vigna radiata*	Salt Temperature Drought	[67,92,93,94]
*bHLH*	*Arabidopsis thaliana* *Oryza sativa* *Vitis vinifera*	Drought Salt Cold Nutrient	[95,96,97]
*NAC*	*Cucumis sativus* *Pyrus ussuriensis* *Miscanthus sinensis*	Salt Drought Cold	[69,98,99]

#### 2.3.2. Translational Regulation

Reported stress responses at the translational level include 5′ ribosomal pauses, translation initiation blockade, and ribosomal changes [1]. In *Arabidopsis*, high-temperature stress induces 5′ ribosomal pausing, leading to mRNA degradation that preferentially affects transcripts encoding HSC/HSP70 chaperone targets [100]. This process is mediated by the extracellular cytoplasmic ribonuclease XRN4 and facilitated by the RNA-binding protein LARP1, a heat-specific cofactor required for XRN4 targeting the polysome [101]. This heat-induced mRNA degradation appears to be required for plant adaptation and survival under chronic heat stress, as dysfunction of XRN4 reduces the heat tolerance of *Arabidopsis* under prolonged exposure to moderately high temperatures [101]. By contrast, XRN4 mediates the mRNA decay of HSFA2, a key regulator of the plant heat stress response, and plants lacking the *AtXRN4* gene function show increased survival upon exposure to short-term severe heat stress, suggesting that heat-induced mRNA degradation plays a negative role in plant resistance to acute heat stress [102].

Under heat stress, translation initiation is blocked, and mRNAs encoding ribosomal proteins (RP) are first stored and sequestered in stress granules. During recovery, these mRNAs are released and translated rapidly to resume translation, a process that is dependent on the chaperone protein HSP101 (also known as CLPB1) [103]. As in the case of the *Arabidopsis* translation initiation factor, mutations in the gene encoding eIF5B result in an inability to adapt to high temperatures; these mutants exhibit delayed multimer recovery in response to high-temperature stress and reduce the translation efficiency of the stress-protected protein subpopulation [104]. In previous research, cold stress increased the expression of the ribosomal biogenesis factor REIL2 [105], and REIL2 overexpression increased cold tolerance in *Arabidopsis*. Conversely, dysfunction of REIL2 resulted in increased sensitivity to cold stress and decreased ribosomal RNA processing and CBF protein levels [105]. Stress responses can also be translated and regulated in the chloroplast. Chilling enhances the binding of the chloroplast RNA-binding protein RBD1 to 23S ribosomal RNA, and RBD1 mutants are defective in the production of mature 23S ribosomal RNA, characterized by the inadequate synthesis of chloroplast proteins, and are highly sensitive to chilling [106].

#### 2.3.3. Post-Translational Regulation

Abiotic stress essentially alters the levels of various types of post-translational modifications (PTMs) that control the localization, accumulation, and activity of proteins and thus play a central role in the regulation of stress responses [1]. The regulation of preexisting signaling proteins by PTMs (including phosphorylation) allows for rapid stress responses, but the stress-induced post-transcriptional de novo synthesis of proteins on PTMs is also important [107]. In addition, PTMs control the activity of many non-signaling proteins that are important for stress resistance. Osmotic stress and ABA signaling lead to the activation of SnRK2s, which include phosphorylate transcription factors, transporter proteins, and many enzymes, including those involved in ROS biogenesis and clearance, as well as osmolyte biosynthesis [108].

ABA strongly activates SnRK2.2, SnRK2.3, and SnRK2.6/OST1 and weakly activates SnRK2.7 and SnRK2.8. SnRK 2.2/3/6 triple mutants in *Arabidopsis* are extremely insensitive to ABA in terms of seed germination, seedling growth, stomatal closure, and gene regulation. Many of the effector proteins of the ABA response are direct substrates of the SnRK2 kinase [3]. bZIP transcription factors such as ABI5 and ABFs (ABA response element-binding factors) are phosphorylated by SnRK2s [16]. Most ABA signaling occurs at the plasma membrane. The association of PYL with the plasma membrane is mediated by its interaction with C2 structural domain proteins [109]. Plasma membrane proteins, such as the anion channel SLAC1, are SnRK2 substrates, which mediate ABA-induced stomatal closure and reduce transpiration water loss under drought stress [3]. Recent phosphoproteomics studies have identified dozens of additional SnRK2 substrate proteins, including several that are critical for chloroplast function, flowering time control, miRNA and chromatin regulation, and RNA splicing [64]. PYL-PP2C-SnRK2 core ABA signaling module activation by MAP3Ks MAP3K17/18, MAP2K MKK3, and MAPKs MPK1/2/7/14 regulates many ABA effector proteins by phosphorylating the MAPK cascade [3].

#### 2.3.4. Epigenetic Regulation

There is growing evidence that epigenetic regulators, particularly histone deacetylases, are involved in the transcriptional regulation of COR genes [110]. In *Arabidopsis*, the histone deacetylase HDA6 is required for cold acclimation and freezing resistance [111]. The histone acetyltransferase GCN5 regulates freezing resistance in *Arabidopsis*. FVE, a WD40 domain-containing protein (also known as WD-repeat protein), functions as a component of the histone deacetylase (HDAC) complex to regulate the cold response. HOS15 is a protein containing a WD40 repeat structural domain that binds to HD2Cs and regulates cold tolerance through histone deacetylation [112]. RNA-directed DNA methylation 4 (RDM4) is important for Pol II-mediated CBF transcription and cold tolerance in *Arabidopsis*. Long non-coding RNAs (lncRNAs) are a group of RNAs that do not encode proteins. Recently, SVALKA, a cold-responsive lncRNA located near the CBF1 locus, was demonstrated to repress CBF1 expression and freezing tolerance [113].

N^6^-methyladenosine (m^6^A) is the most abundant internal chemical modification in eukaryotic mRNA. In recent years, a large body of evidence has demonstrated that it plays vital roles in plant abiotic stresses [114,115]. Studies have found that the expression level of the RNA m^6^A methyltransferase components, MTA, MTB, FIP37, and VIR, was increased during salt stress [114]. Hou et al. found that MdMTA was involved in the lignin metabolic process and oxidative stress under drought conditions [116]. In *Arabidopsis*, RNA demethylase ALKBH9B and ALKBH10B were demonstrated to modulate ABA response via regulating the mRNA m^6^A level [117,118]. Furthermore, ALKBH10B can also be induced by salt and osmotic stress [117,119]. In addition, the IYT521-B homology (YTH) domain proteins were induced by various abiotic stress and phytohormones [120]. Interestingly, the cytoplasmic protein ECT2 has been reported to relocalize to stress granules in response to heat stress [121]. These findings suggested that epigenetic regulation was important for plant response to abiotic stress.

## 3. Strategies to Improve Plant Growth under Stress

### 3.1. Natural Genetic Variation

In recent years, quantitative trait loci and genome-wide association studies have increasingly identified important regulators and natural allelic variants of crop responses to abiotic stresses. Using these approaches, identified genes and alleles can be used to produce more adaptive and higher yielding crops. For example, *HKT1* alleles in rice, wheat (*Triticum aestivum*), and maize (*Zea mays*) have been identified as key quantitative trait loci that control salt tolerance in plants and lead to increased yields in wheat when grown with markers on saline soils [122,123]. Furthermore, in the African rice subspecies *Oryza glaberrima*, temperature tolerance 1 (TT1) has been identified as a key quantitative trait locus controlling temperature tolerance and plays an important role in local adaptation [124]. Genome-wide association studies have indicated that genetic variation in the Na^+^ and K^+^ transporter gene *SlHAK20* was responsible for changes in the Na^+^/K^+^ ratio in tomato roots and the loss of salt tolerance during tomato domestication [125]. In addition, genome-wide association analysis has shown that natural variation in the tomato SOS1 gene (*SlSOS1*) contributes to phenotypic changes in salt tolerance in tomato, whereas genetic variation in the genes encoding the vacuolar H^+^ pyrophosphatase ZmVPP1 and the NAC transcription factor ZmNAC111 contributes to drought tolerance in maize seedlings [126]. As an example of crop improvement using identified stress regulators, transgenic maize with increased expression of ZmVPP1 showed better drought tolerance, possibly due to increased photosynthetic efficiency and root development [127].

### 3.2. Genetic Engineering

Resistant plants can be genetically modified through increasing or decreasing the expression or activity of key regulators involved in the resistance response. Although regulators can be manipulated at any molecular level of the response, protein kinases and other signaling components, transcription factors, metabolic enzymes, and ion transporters have been the most successful [1]. For example, transgenic rice plants expressing the stress-inducible *OsDREB2A* showed greater tolerance to dehydration stress [128]. The excessive accumulation of the cytotoxic metabolite methylglyoxal (MG) frequently occurs under stress conditions. The overexpression of genes in the glyoxalase detoxification pathway can increase tolerance to abiotic stresses including high salinity, drought, and high temperature. Mock knockdown of *miR166* in rice using short tandem targets increased drought tolerance and induced developmental changes that mimicked the natural plant response to water stress, such as reduced leaf curl and xylem diameter [129]. In addition to plant genes, some microbial genes, such as the RNA-stabilizing *Bacillus subtilis* cold shock protein B, have been used to develop commercial stress-tolerant transgenes. The overexpression of the osmotic stress-activated and ABA-activated SnRK2 genes *SAPK1* and *SAPK2* increased salt tolerance in *Oryza* spp. [130]. Loss-of-function mutants of these genes generated using CRISPR-Cas genome editing showed reduced salt tolerance, confirming the important role of SAPK1 and SAPK2 in this process [130]. CRISPR-Cas-based gene editing is a powerful genetic engineering technique that can create random small indel mutations or precise base changes through base editing, plasmid editing, and targeted sequence insertion and substitution during culture [131,132]. In particular, targeted sequence insertion technology can be used to efficiently introduce transcriptional or translational regulatory sequences into key stress-responsive genes to produce alleles with increased or decreased expression, making it a valuable tool for research and breeding [133].

### 3.3. Chemical Intervention

Abiotic stresses are considered major factors in the reduction of plant yield and quality. Plants survive and adapt to adversity through slowing growth and development, while increased plant resistance inhibits plant growth [3]. It is therefore particularly important to develop effective strategies to mitigate abiotic stresses. Recent studies have shown that the exogenous pretreatment of plants with chemical agents can induce existing molecular and physiological defense mechanisms in plants to enhance abiotic stress tolerance in a process that is known as chemical priming [134]. Chemical priming is considered to be a promising strategy for enhancing plant stress tolerance, as it allows plants to rapidly activate abiotic stress responses when exposed to stressful conditions, which is more conducive to plant survival. Moreover, chemical priming is functionally diverse, can enhance tolerance to many types of abiotic stresses, and is applicable to many plant species.

When plants are subjected to abiotic stresses, endogenous hormones are altered to regulate the relevant physiological and biochemical processes. For example, ABA plays an integral role in plant responses to abiotic stresses, with stresses such as drought, high salinity, and low temperature inducing ABA synthesis. ABA is known to be involved in regulating several plant physiological and biochemical processes, including altering stomatal resistance to regulate the leaf transpiration rate, promoting the synthesis and accumulation of osmotically stressed substances such as proline, and regulating seed and shoot dormancy [110]. In addition, the exogenous application of ABA has been reported to enhance stress resistance in plants; that is, plant stress resistance can also be regulated by exogenous treatment with small molecule compounds associated with the activity of molecular components of the stress response network [1]. Studies have shown that ABA is an effective starter hormone that enhances tolerance to various stresses in a range of crops [135,136,137,138,139]. In rice, for example, exogenous ABA increased the tolerance of four rice genotypes to low-temperature stress, enhanced the capacity of the antioxidant defense system, and reduced the damage caused by ROS [138]. Moreover, exogenous ABA pretreatment improved the resistance of rice seedlings to alkaline stress, reduced seedling root damage, and increased survival and grain yield [139]. This suggests that ABA effectively initiates tolerance to saline stress in rice plants. In addition, ABA has achieved significant results in improving the drought tolerance of plants. The application of exogenous ABA promoted stomatal closure and reduced transpiration to control water use, which helped to enhance tolerance to drought stress [139].

### 3.4. Transcriptome Analyses (RNA-Seq)

Transcriptome analysis has become increasingly important and common in plant stress resistance studies because of the importance of genes in the response to abiotic stresses and the rapid development of RNA-seq technology. Plants exhibit the differential expression of a number of functional and regulatory genes in response to abiotic stresses, forming a complex regulatory network. Transcriptome sequencing technology can quantify gene expression and uncover important functional genes and is therefore an important research tool in the study of abiotic stresses. Although different stress response mechanisms vary, transcriptomic technologies are generally similar in their approach to research, with the key being to explore the regulatory mechanisms underlying differences in stress resistance. First, through pre-experimentation, samples are taken and sequenced at time points at which phenotypic changes are evident following abiotic stress treatment; this is followed by principal component analysis (PCA), which looks at different materials as a whole and identifies sample-to-sample variability. It is important to screen for abiotic stress-related candidate genes and to validate the reliability of the transcriptome data. In stress resistance studies, combined broad-target metabolome and transcriptome analyses are often used to target major metabolic pathways and associated genes, and to screen for key regulators, helping to accelerate the resolution of plant stress-response mechanisms.

For example, in the study of temperature stress, low-temperature and high-temperature differential genes were screened using transcriptomics, and the differential genes were further investigated. The results showed that oilseed rape obtained higher cold tolerance by upregulating the expression of photosynthetic genes; by contrast, high-temperature stress suppressed the expression of key genes and weakened the plant’s ability to self-regulate [140]. Furthermore, transcriptome sequencing and comparative analysis of drought-tolerant (N22) and drought-sensitive (IR64) rice plants revealed several key genes known to be involved in drought stress, whereas 1366 differential genes were found to exhibit completely opposite regulatory patterns in the two rice varieties under similar drought conditions [141]. In the study of stress mechanisms in plants, there is also the problem of a lack of high-quality genomes, which prevents in-depth studies. Therefore, researchers have used the combined analysis of triple sequencing and second-generation sequencing data to obtain valuable reference data and the key genes involved in abiotic stress response [142,143,144]. In summary, studies of stress often take the form of exploring “differences,” combining transcriptomic techniques to find key nodes or genes for stress resistance at the level of transcriptional regulation and then combining these findings with multi-omics analysis or follow-up studies.

## 4. Future Perspectives

In the wild, plants constantly face different forms of stress. The ideal growth conditions for any plant can only be achieved in a controlled setting. Increasing evidence indicates that the trade-off is primarily caused by the active suppression of growth via stress signaling pathways [3]. At several levels, the regulatory networks for controlling the stress response and growth interact. Further studies will clarify key relationships and offer strategies for boosting stress resistance with little or no yield cost.

There is still much scope for research into the molecular response mechanisms of plants to abiotic stresses. The identification of stress sensors remains an important but challenging goal in plant abiotic stress research. Efficient gene editing techniques and chemical genetic approaches can help overcome the genetic overload that hinders the genetic identification of stress sensors. Further understanding of the roles of different organelles in stress perception and response, as well as dispersion models of stress perception, will also contribute to the understanding of stress perception and stress tolerance, although the integration of signals from damaged organelles remains poorly understood.

The signaling pathways of stress transduction are intricate and complex, and even as researchers continue to enrich knowledge in this field through combining genomics, transcriptomics, metabolomics, genetics, and other approaches, there are still challenges beyond our reach to fully elucidate the complete regulatory network of responses. In the natural environment, plants are exposed to a wide range of environmental factors due to their immobile nature, making it crucial to understand the cross-linked interactions between common stress response pathways. Moreover, there is still great potential to study biotic and abiotic stresses in combination. In the future, multidisciplinary efforts are needed to unravel the integrated regulation of plant responses to multiple stresses, which is of great importance for crop breeding and production.

## Data Availability

Not applicable.

## References

[1] Zhang H., Zhu J., Gong Z., Zhu J.-K. (2022). Abiotic stress responses in plants. Nat. Rev. Genet..

[2] Zhang H., Liu S., Ren T., Niu M., Liu X., Liu C., Wang H., Yin W., Xia X. (2023). Crucial abiotic stress regulatory network of NF-Y transcription factor in plants. Int. J. Mol. Sci..

[3] Zhang H., Zhao Y., Zhu J.-K. (2020). Thriving under stress: How plants balance growth and the stress response. Dev. Cell.

[4] Swain R., Sahoo S., Behera M., Rout G.R. (2023). Instigating prevalent abiotic stress resilience in crop by exogenous application of phytohormones and nutrient. Front. Plant Sci..

[5] Markham K.K., Greenham K. (2021). Abiotic stress through time. New Phytol..

[6] Praveen A., Dubey S., Singh S., Sharma V.K. (2023). Abiotic stress tolerance in plants: A fascinating action of defense mechanisms. 3 Biotech.

[7] Beena R., Sunitha N.C., Sah R.P., Krishna G.K., Umesh D., Thomas S., Anilkumar C., Upadhyay S., Kumar A., Manikanta C.L.N. (2023). Physiological and molecular implications of multiple abiotic stresses on yield and quality of rice. Front. Plant Sci..

[8] Dong Q., Wallrad L., Almutairi B.O., Kudla J. (2022). Ca^2+^ signaling in plant responses to abiotic stresses. J. Integr. Plant Biol..

[9] Munns R., Passioura J.B., Colmer T.D., Byrt C.S. (2020). Osmotic adjustment and energy limitations to plant growth in saline soil. New Phytol..

[10] Gong Z., Xiong L., Shi H., Yang S., Herrera-Estrella L.R., Xu G., Chao D.Y., Li J., Wang P.Y., Qin F. (2020). Plant abiotic stress response and nutrient use efficiency. Sci. China Life Sci..

[11] Alpert P. (2006). Constraints of tolerance: Why are desiccation-tolerant organisms so small or rare?. J. Exp. Biol..

[12] Carpenter J.F., Crowe J.H., Arakawa T. (1990). Comparison of solute-induced protein stabilization in aqueous solution and in the frozen and dried states. J. Dairy Sci..

[13] Kishor P.B.K., Sangam S., Amrutha R.N., Laxmi P.S., Naidu K.R., Rao K.R.S.S., Rao S., Reddy K.J., Sreenivasulu N. (2004). Regulation of proline biosynthesis, degradation, uptake and transport in higher plants: Its implications in plant growth and abiotic stress tolerance. Curr. Sci..

[14] Maity K., Heumann J.M., McGrath A.P., Kopcho N.J., Hsu P.-K., Lee C.-W., Mapes J.H., Garza D., Krishnan S., Morgan G.P. (2019). Cryo-EM structure of OSCA1.2 from *Oryza sativa* elucidates the mechanical basis of potential membrane hyperosmolality gating. Proc. Natl. Acad. Sci. USA.

[15] Liu X., Wang J., Sun L. (2018). Structure of the hyperosmolality-gated calcium-permeable channel OSCA1.2. Nat. Commun..

[16] Maszkowska J., Szymańska K.P., Kasztelan A., Krzywińska E., Sztatelman O., Dobrowolska G. (2021). The multifaceted regulation of SnRK2 kinases. Cells.

[17] Hou Q., Ufer G., Bartels D. (2016). Lipid signalling in plant responses to abiotic stress. Plant Cell Environ..

[18] Gatica D., Hu G., Zhang N., Williamson P.R., Klionsky D.J. (2019). The Pat1-Lsm complex prevents 3′ to 5′ degradation of a specific subset of *ATG* mRNAs during nitrogen starvation-induced autophagy. Autophagy.

[19] Nakamura S., Masanori I. (2019). Chlorophagy is *ATG* gene-dependent microautophagy process. Plant Signal. Behav..

[20] Young P.G., Passalacqua M.J., Chappell K., Llinas R.J., Bartel B. (2019). A facile forward-genetic screen for *Arabidopsis* autophagy mutants reveals twenty-one loss-of-function mutations disrupting six *ATG* genes. Autophagy.

[21] Izumi M., Ishida H., Nakamura S., Hidema J. (2017). Entire photodamaged chloroplasts are transported to the central vacuole by autophagy. Plant Cell.

[22] Lystad A.H., Simonsen A. (2019). Mechanisms and pathophysiological roles of the ATG8 conjugation machinery. Cells.

[23] Nguyen T.N., Padman B.S., Usher J., Oorschot V., Ramm G., Lazarou M. (2016). ATG8 family LC3/GABARAP proteins are crucial for autophagosome–lysosome fusion but not autophagosome formation during PINK1/Parkin mitophagy and starvation. J. Cell Biol..

[24] Zhu J.-K. (2002). Salt and drought stress signal transduction in plants. Annu. Rev. Plant Biol..

[25] Lizana C., Wentworth M., Martinez J.P., Villegas D., Meneses R., Murchie E.H., Pastenes C., Lercari B., Vernieri P., Horton P. (2006). Differential adaptation of two varieties of common bean to abiotic stress: I. Effects of drought on yield and photosynthesis. J. Exp. Bot..

[26] Zhang Y., Kaiser E., Li T., Marcelis L.F.M. (2022). NaCl affects photosynthetic and stomatal dynamics by osmotic effects and reduces photosynthetic capacity by ionic effects in tomato. J. Exp. Bot..

[27] Zhang Y., Ge Y.R., Zhao R., Hu Y.T., Chen Y., Guo Y.Y., Lin J.X., Li R.L. (2022). Progress on the structural components, biosynthesis and functions of suberin. Chin. Sci. Bull..

[28] Qiu Q.-S., Guo Y., Dietrich M.A., Schumaker K.S., Zhu J.-K. (2002). Regulation of SOS1, a plasma membrane Na^+^/H^+^ exchanger in *Arabidopsis thaliana* by SOS2 and SOS3. Proc. Natl. Acad. Sci. USA.

[29] Qiu Q.-S., Guo Y., Quintero F.J., Pardo J.M., Schumaker K.S., Zhu J.-K. (2004). Regulation of vacuolar Na^+^/H^+^ exchange in *Arabidopsis thaliana* by the salt-overly-sensitive (SOS) pathway. J. Biol. Chem..

[30] Zhu J.-K. (2016). Abiotic stress signaling and responses in plants. Cell.

[31] Zhang H., Wang Z., Li X., Gao X., Dai Z., Cui Y., Zhi Y., Liu Q., Zhai H., Gao S. (2022). The IbBBX24–IbTOE3–IbPRX17 module enhances abiotic stress tolerance by scavenging reactive oxygen species in sweet potato. New Phytol..

[32] Chinnusamy V., Zhu J., Zhu J.-K. (2007). Cold stress regulation of gene expression in plants. Trends Plant Sci..

[33] Sangwan V., Örvar B.L., Beyerly J., Hirt H., Dhindsa R.S. (2002). Opposite changes in membrane fluidity mimic cold and heat stress activation of distinct plant map kinase pathways. Plant J..

[34] Manasa S.L., Panigrahy M., Panigrahi K.C.S., Rout G.R. (2022). Overview of cold stress regulation in plants. Bot. Rev..

[35] Liu Q., Ding Y., Shi Y., Ma L., Wang Y., Song C., Wilkins K.A., Davies J.M., Knight H., Knight M.R. (2021). The calcium transporter ANNEXIN1 mediates cold-induced calcium signaling and freezing tolerance in plants. EMBO J..

[36] Furuya T., Matsuoka D., Nanmori T. (2013). Phosphorylation of *Arabidopsis thaliana* MEKK1 via Ca²^+^ signaling as a part of the cold stress response. J. Plant Res..

[37] Rubab S., Talha J., Sadam H., Sunny A., Misbah N., Hina Z., Saurabh P., Jyoti C., Manzer H.S., Chen P. (2022). Calcium homeostasis and potential roles to combat environmental stresses in plants. S. Afr. J. Bot..

[38] Jiang B., Shi Y., Peng Y., Jia Y., Yan Y., Dong X., Li H., Dong J., Li J., Gong Z. (2020). Cold-induced CBF–PIF3 interaction enhances freezing tolerance by stabilizing the phyB thermosensor in *Arabidopsis*. Mol. Plant.

[39] Scharf K.-D., Berberich T., Ebersberger I., Nover L. (2012). The plant heat stress transcription factor (Hsf) family: Structure, function and evolution. BBA-Gene Regul. Mech..

[40] Lin B.-L., Wang J.-S., Liu H.-C., Chen R.-W., Meyer Y., Barakat A., Delseny M. (2001). Genomic analysis of the Hsp70 superfamily in *Arabidopsis thaliana*. Cell Stress Chaperones.

[41] Wang W., Vinocur B., Shoseyov O., Altman A. (2004). Role of plant heat-shock proteins and molecular chaperones in the abiotic stress response. Trends Plant Sci..

[42] Li J., Liu X. (2019). Genome-wide identification and expression profile analysis of the *Hsp20* gene family in barley (*Hordeum vulgare* L.). PeerJ.

[43] Siddique M., Gernhard S., von Koskull-Doring P., Vierling E., Scharf K.D. (2008). The plant sHSP superfamily: Five new members in *Arabidopsis thaliana* with unexpected properties. Cell Stress Chaperones.

[44] Jung K.-H., Gho H.-J., Nguyen M.X., Kim S.-R., An G. (2013). Genome-wide expression analysis of *HSP70* family genes in rice and identification of a cytosolic *HSP70* gene highly induced under heat stress. Funct. Integr. Genom..

[45] Song J., Ma H., Weng Q., Yuan J., Zhao Y., Liu Y. (2017). Genome-wide identification and analysis of *HSP70* gene family in *Maize*. J. Nucl. Agric. Sci..

[46] Augustine S.M., Cherian A.V., Syamaladevi D.P., Subramonian N. (2015). *Erianthus arundinaceus* HSP70 (EaHSP70) acts as a key regulator in the formation of anisotropic interdigitation in sugarcane (*Saccharum* spp. hybrid) in response to drought stress. Plant Cell Physiol..

[47] Guo M. (2016). Expression Analysis and Functional Study of Heat Stress Related Genes in Pepper. Ph.D. Thesis.

[48] Zhao X., Zhang T., Xing W., Wang J., Song X., Zhou Y. (2021). Genome-wide identification and expression analysis under temperature stress of *HSP70* gene family in *Dendrobium catenatum*. Acta Hortic..

[49] Park C.J., Seo Y.S. (2015). Heat shock proteins: A review of the molecular chaperones for plant immunity. Plant Pathol. J..

[50] Du Q., Jiang J., Chen M., Ning N., Reng M., Li X., Xie X. (2021). Cloning, expression analysis, and prokaryotic expression of rice heat shock protein *HSP70* gene. J. Plant Protec..

[51] Martí M.C., Stancombe M.A., Webb A.A.R. (2013). Cell- and stimulus type-specific intracellular free Ca^2+^ signals in *Arabidopsis*. Plant Physiol..

[52] Brandt B., Munemasa S., Wang C., Nguyen D., Yong T., Yang P.G., Poretsky E., Belknap T.F., Waadt R., Alemán F. (2015). Calcium specificity signaling mechanisms in abscisic acid signal transduction in *Arabidopsis* guard cells. eLife.

[53] Hrabak E.M., Chan C.W., Gribskov M., Harper J.F., Choi J.H., Halford N., Kudla J., Luan S., Nimmo H.G., Sussman M.R. (2003). The *Arabidopsis* CDPK-SnRK superfamily of protein kinases. Plant Physiol..

[54] Ma L., Ye J., Yang Y., Lin H., Yue L., Luo J., Long Y., Fu H., Liu X., Zhang Y. (2019). The SOS2-SCaBP8 complex generates and fine-tunes an AtANN4-dependent calcium signature under salt stress. Dev. Cell.

[55] Qi J., Song C.-P., Wang B., Zhou J., Kangasjärvi J., Zhu J.-K., Gong Z. (2018). Reactive oxygen species signaling and stomatal movement in plant responses to drought stress and pathogen attack. J. Integr. Plant Biol..

[56] Min M.K., Choi E.-H., Kim J.-A., Yoon I.S., Han S., Lee Y., Lee S., Kim B.-G. (2019). Two clade a phosphatase 2Cs expressed in guard cells physically interact with abscisic acid signaling components to induce stomatal closure in rice. Rice.

[57] Geiger D., Scherzer S., Mumm P., Marten I., Ache P., Matschi S., Liese A., Wellmann C., Al-Rasheid K.A., Grill E. (2010). Guard cell anion channel SLAC1 is regulated by CDPK protein kinases with distinct Ca^2+^ affinities. Proc. Natl. Acad. Sci. USA.

[58] Geiger D., Scherzer S., Mumm P., Stange A., Marten I., Bauer H., Ache P., Matschi S., Liese A., Al-Rasheid K.A.S. (2009). Activity of guard cell anion channel SLAC1 is controlled by drought-stress signaling kinase-phosphatase pair. Proc. Natl. Acad. Sci. USA.

[59] Wang P., Hsu C.-C., Du Y., Zhu P., Zhao C., Fu X., Zhang C., Paez J.S., Macho A.P., Tao W.A. (2020). Mapping proteome-wide targets of protein kinases in plant stress responses. Proc. Natl. Acad. Sci. USA.

[60] Yang T., Shad Ali G., Yang L., Du L., Reddy A.S.N., Poovaiah B.W. (2010). Calcium/calmodulin-regulated receptor-like kinase CRLK1 interacts with MEKK1 in plants. Plant Signal. Behav..

[61] Zhao C., Wang P., Si T., Hsu C.-C., Wang L., Zayed O., Yu Z., Zhu Y., Dong J., Tao W.A. (2017). Map kinase cascades regulate the cold response by modulating ICE1 protein stability. Dev. Cell.

[62] Ma Y., Szostkiewicz I., Korte A., Moes D., Yang Y., Christmann A., Grill E. (2009). Regulators of PP2C phosphatase activity function as abscisic acid sensors. Science.

[63] Melcher K., Ng L.-M., Zhou X.E., Soon F.-F., Xu Y., Suino-Powell K.M., Park S.-Y., Weiner J.J., Fujii H., Chinnusamy V. (2009). A gate–latch–lock mechanism for hormone signalling by abscisic acid receptors. Nature.

[64] Wang P., Xue L., Batelli G., Lee S., Hou Y.-J., Van Oosten M.J., Zhang H., Tao W.A., Zhu J.-K. (2013). Quantitative phosphoproteomics identifies SnRK2 protein kinase substrates and reveals the effectors of abscisic acid action. Proc. Natl. Acad. Sci. USA.

[65] Park S.-Y., Fung P., Nishimura N., Jensen D.R., Fujii H., Zhao Y., Lumba S., Santiago J., Rodrigues A., Chow T.-f.F. (2009). Abscisic acid inhibits type 2C protein phosphatases via the PYR/PYL family of start proteins. Science.

[66] Nag M., Lahiri D., Garai S., Mukherjee D., Ray R.R. (2021). Regulation of β-amylase synthesis: A brief overview. Mol. Biol. Rep..

[67] Banerjee A., Roychoudhury A. (2017). Abscisic-acid-dependent basic leucine zipper (bZIP) transcription factors in plant abiotic stress. Protoplasma.

[68] Jang Y.-H., Park J.-R., Kim E.-G., Kim K.-M. (2022). *OsbHLHq11*, the basic helix-loop-helix transcription factor, involved in regulation of chlorophyll content in rice. Biology.

[69] Yang X., He K., Chi X., Chai G., Wang Y., Jia C., Zhang H., Zhou G., Hu R. (2018). *Miscanthus* NAC transcription factor MlNAC12 positively mediates abiotic stress tolerance in transgenic *Arabidopsis*. Plant Sci..

[70] Ma S., Bohnert H.J. (2007). Integration of *Arabidopsis thaliana* stress-related transcript profiles, promoter structures, and cell-specific expression. Genome Biol..

[71] Narusaka Y., Nakashima K., Shinwari Z.K., Sakuma Y., Furihata T., Abe H., Narusaka M., Shinozaki K., Yamaguchi-Shinozaki K. (2003). Interaction between two *cis*-acting elements, ABRE and DRE, in ABA-dependent expression of *Arabidopsis rd29A* gene in response to dehydration and high-salinity stresses. Plant J..

[72] Yamaguchi-Shinozaki K., Shinozaki K. (2006). Transcriptional regulatory networks in cellular responses and tolerance to dehydration and cold stresses. Annu. Rev. Plant Biol..

[73] Lee D.K., Kim H.I., Jang G., Chung P.J., Jeong J.S., Kim Y.S., Bang S.W., Jung H., Choi Y.D., Kim J.K. (2015). The NF-YA transcription factor OsNF-YA7 confers drought stress tolerance of rice in an abscisic acid independent manner. Plant Sci..

[74] Hwang K., Susila H., Nasim Z., Jung J.-Y., Ahn J.H. (2019). *Arabidopsis* ABF3 and ABF4 transcription factors act with the NF-YC complex to regulate *SOC1* expression and mediate drought-accelerated flowering. Mol. Plant.

[75] Quach T.N., Nguyen H.T., Valliyodan B., Joshi T., Xu D., Nguyen H.T. (2015). Genome-wide expression analysis of soybean NF-Y genes reveals potential function in development and drought response. Mol. Genet. Genom..

[76] Li S., Zhang N., Zhu X., Ma R., Liu S., Wang X., Yang J., Si H. (2021). Genome-wide analysis of *NF-Y* genes in potato and functional identification of *StNF-YC9* in drought tolerance. Front. Plant Sci..

[77] Yang Y., Wang B., Wang J., He C., Zhang D., Li P., Zhang J., Li Z. (2022). Transcription factors ZmNF-YA1 and ZmNF-YB16 regulate plant growth and drought tolerance in maize. Plant Physiol..

[78] Feng S.-S., Wang L., Zhou Y., Wang Y.-P., Fang Y.-J. (2023). Research progresses on *WOX* family genes in regulating plant development and abiotic stress response. Biotechnol. Bull..

[79] Minh-Thu P.T., Kim J.S., Chae S., Jun K.M., Lee G.S., Kim D.E., Cheong J.J., Song S.I., Nahm B.H., Kim Y.K. (2018). A WUSCHEL homeobox transcription factor, OsWOX13, enhances drought tolerance and triggers early flowering in rice. Mol. Cells.

[80] Fambrini M., Usai G., Pugliesi C. (2022). Induction of somatic embryogenesis in plants: Different players and focus on WUSCHEL and WUS-related homebox (WOX) transcription factors. Int. J. Mol. Sci..

[81] Sajjad M., Wei X., Liu L., Li F., Ge X. (2021). Transcriptome analysis revealed GhWOX4 intercedes myriad regulatory pathways to modulate drought tolerance and vascular growth in cotton. Int. J. Mol. Sci..

[82] Shafique Khan F., Zeng R.F., Gan Z.M., Zhang J.Z., Hu C.G. (2021). Genome-wide identification and expression profiling of the *WOX* gene family in *Citrus sinensis* and functional analysis of a *CsWUS* Member. Int. J. Mol. Sci..

[83] Haake V., Cook D., Riechmann J.L., Pineda O., Thomashow M.F., Zhang J.Z. (2002). Transcription factor CBF4 is a regulator of drought adaptation in *Arabidopsis*. Plant Physiol..

[84] Chen G., Feng H., Hu Q., Qu H., Chen A., Yu L., Xu G. (2015). Improving rice tolerance to potassium deficiency by enhancing *OsHAK16p:WOX11*-controlled root development. Plant Biotechnol. J..

[85] Charvin M., Halter T., Blanc-Mathieu R., Barraud P., Aumont-Nicaise M., Parcy F., Navarro L. (2023). Single-cytosine methylation at W-boxes repels binding of WRKY transcription factors through steric hindrance. Plant Physiol..

[86] Ma Y., Xue H., Zhang F., Jiang Q., Yang S., Yue P., Wang F., Zhang Y., Li L., He P. (2021). The miR156/SPL module regulates apple salt stress tolerance by activating MdWRKY100 expression. Plant Biotechnol. J..

[87] Shikha K., Madhumal Thayil V., Shahi J.P., Zaidi P.H., Seetharam K., Nair S.K., Singh R., Tosh G., Singamsetti A., Singh S. (2023). Genomic-regions associated with cold stress tolerance in Asia-adapted tropical maize germplasm. Sci. Rep..

[88] Liu Y., Yang T., Lin Z., Gu B., Xing C., Zhao L., Dong H., Gao J., Xie Z., Zhang S. (2019). A WRKY transcription factor PbrWRKY53 from *Pyrus betulaefolia* is involved in drought tolerance and AsA accumulation. Plant Biotechnol. J..

[89] Lee F.C., Yeap W.C., Appleton D.R., Ho C.L., Kulaveerasingam H. (2022). Identification of drought responsive *Elaeis guineensis* WRKY transcription factors with sensitivity to other abiotic stresses and hormone treatments. BMC Genom..

[90] Zandalinas S.I., Mittler R., Balfagón D., Arbona V., Gómez-Cadenas A. (2018). Plant adaptations to the combination of drought and high temperatures. Physiol. Plant.

[91] Gémes K., Mellidou Ι., Karamanoli K., Beris D., Park K.Y., Matsi T., Haralampidis K., Constantinidou H.I., Roubelakis-Angelakis K.A. (2017). Deregulation of apoplastic polyamine oxidase affects development and salt response of tobacco plants. J. Plant Physiol..

[92] Zhang W., Ye S., Du Y., Zhao Q., Du J., Zhang Q. (2022). Identification and expression analysis of bZIP members under abiotic stress in mung bean (*Vigna radiata*). Life.

[93] Agarwal P., Baranwal V.K., Khurana P. (2019). Genome-wide analysis of bZIP transcription factors in wheat and functional characterization of a *TabZIP* under abiotic stress. Sci. Rep..

[94] Manzoor M.A., Manzoor M.M., Li G., Abdullah M., Han W., Wenlong H., Shakoor A., Riaz M.W., Rehman S., Cai Y. (2021). Genome-wide identification and characterization of bZIP transcription factors and their expression profile under abiotic stresses in Chinese pear (*Pyrus bretschneideri*). BMC Plant Biol..

[95] Le Hir R., Castelain M., Chakraborti D., Moritz T., Dinant S., Bellini C. (2017). AtbHLH68 transcription factor contributes to the regulation of ABA homeostasis and drought stress tolerance in *Arabidopsis thaliana*. Physiol. Plant.

[96] Alsamman A.M., Abdelsattar M., El Allali A., Radwan K.H., Nassar A.E., Mousa K.H., Hussein A., Mokhtar M.M., Abd El-Maksoud M.M., Istanbuli T. (2023). Genome-wide identification, characterization, and validation of the bHLH transcription factors in grass pea. Front. Genet..

[97] Wang F., Itai R.N., Nozoye T., Kobayashi T., Nishizawa N.K., Nakanishi H. (2020). The bHLH protein OsIRO3 is critical for plant survival and iron (Fe) homeostasis in rice (*Oryza sativa* L.) under Fe-deficient conditions. Soil Sci. Plant Nutr..

[98] Zhang X., Yu H., Sun C., Deng J., Zhang X., Liu P., Li Y., Li Q., Jiang W. (2017). Genome-wide characterization and expression profiling of the *NAC* genes under abiotic stresses in *Cucumis sativus*. Plant Physiol. Biochem..

[99] Ahmad M., Alabd A., Gao Y., Yu W., Jamil W., Wang X., Wei J., Ni J., Teng Y., Bai S. (2022). Three stress-responsive NAC transcription factors, Pp-SNACs, differentially and synergistically regulate abiotic stress in pear. Sci. Hortic..

[100] Merret R., Nagarajan V.K., Carpentier M.-C., Park S., Favory J.-J., Descombin J., Picart C., Charng Y.-y., Green P.J., Deragon J.-M. (2015). Heat-induced ribosome pausing triggers mRNA co-translational decay in *Arabidopsis thaliana*. Nucleic Acids Res..

[101] Merret R., Descombin J., Juan Y.-t., Favory J.-J., Carpentier M.-C., Chaparro C., Charng Y.-y., Deragon J.-M., Bousquet-Antonelli C. (2013). XRN4 and LARP1 are required for a heat-triggered mRNA decay pathway involved in plant acclimation and survival during thermal stress. Cell Rep..

[102] Nguyen A.H., Matsui A., Tanaka M., Mizunashi K., Nakaminami K., Hayashi M., Iida K., Toyoda T., Nguyen D.V., Seki M. (2015). Loss of *Arabidopsis* 5′–3′ exoribonuclease AtXRN4 function enhances heat stress tolerance of plants subjected to severe heat stress. Plant Cell Physiol..

[103] Merret R., Carpentier M.-C., Favory J.-J., Picart C., Descombin J., Bousquet-Antonelli C., Tillard P., Lejay L., Deragon J.-M., Charng Y.-y. (2017). Heat shock protein Hsp101 affects the release of ribosomal protein mRNAs for recovery after heat shock. Plant Physiol..

[104] Chukka P.A.R., Wetmore S.D., Thakor N. (2021). Established and emerging regulatory roles of eukaryotic translation initiation factor 5B (eIF5B). Front. Genet..

[105] Yu H., Kong X., Huang H., Wu W., Park J., Yun D.-J., Lee B.-H., Shi H., Zhu J.-K. (2020). STCH4/REIL2 confers cold stress tolerance in *Arabidopsis* by promoting rRNA processing and CBF protein translation. Cell Rep..

[106] Nishimura K., Ashida H., Ogawa T., Yokota A. (2010). A DEAD box protein is required for formation of a hidden break in *Arabidopsis* chloroplast 23S rRNA. Plant J..

[107] Ding Y., Lv J., Shi Y., Gao J., Hua J., Song C., Gong Z., Yang S. (2018). EGR2 phosphatase regulates OST1 kinase activity and freezing tolerance in *Arabidopsis*. EMBO J..

[108] Willems P., Horne A., Van Parys T., Goormachtig S., De Smet I., Botzki A., Van Breusegem F., Gevaert K. (2019). The plant PTM viewer, a central resource for exploring plant protein modifications. Plant J..

[109] Rodrigues A., Adamo M., Crozet P., Margalha L., Confraria A., Martinho C., Elias A., Rabissi A., Lumbreras V., González-Guzmán M. (2013). ABI1 and PP2CA phosphatases are negative regulators of Snf1-related protein kinase1 signaling in *Arabidopsis*. Plant Cell.

[110] Chauhan D.K., Yadav V., Vaculík M., Gassmann W., Pike S., Arif N., Singh V.P., Deshmukh R., Sahi S., Tripathi D.K. (2021). Aluminum toxicity and aluminum stress-induced physiological tolerance responses in higher plants. Crit. Rev. Biotechnol..

[111] To T.K., Nakaminami K., Kim J.-M., Morosawa T., Ishida J., Tanaka M., Yokoyama S., Shinozaki K., Seki M. (2011). *Arabidopsis* HDA6 is required for freezing tolerance. Biochem. Biophys. Res. Commun..

[112] Park J., Lim C.J., Shen M., Park H.J., Cha J.-Y., Iniesto E., Rubio V., Mengiste T., Zhu J.-K., Bressan R.A. (2018). Epigenetic switch from repressive to permissive chromatin in response to cold stress. Proc. Natl. Acad. Sci. USA.

[113] Kindgren P., Ard R., Ivanov M., Marquardt S. (2018). Transcriptional read-through of the long non-coding RNA svalka governs plant cold acclimation. Nat. Commun..

[114] Yue Y., Liu J., He C. (2015). RNA N6-methyladenosine methylation in post-transcriptional gene expression regulation. Genes Dev..

[115] Hu J., Cai J., Park S.J., Lee K., Li Y., Chen Y., Yun J.Y., Xu T., Kang H. (2021). N6-methyladenosine mRNA methylation is important for salt stress tolerance in *Arabidopsis*. Plant J..

[116] Hou N., Li C., He J., Liu Y., Yu S., Malnoy M., Mobeen T.M., Xu L., Ma F., Guan Q. (2022). MdMTA-mediated m^6^A modification enhances drought tolerance by promoting mRNA stability and translation efficiency of genes involved in lignin deposition and oxidative stress. New Phytol..

[117] Shoaib Y., Hu J., Manduzio S., Kang H. (2021). Apha-ketoglutarate-dependent dioxygenase homolog 10B, an N6-methyladenosine mRNA demethylase, plays a role in salt stress and abscisic acid responses in *Arabidopsis thaliana*. Physiol. Plant.

[118] Tang J., Yang J., Duan H., Jia G. (2021). ALKBH10B, an mRNA m^6^A demethylase, modulates ABA response during seed germination in *Arabidopsis*. Front. Plant. Sci..

[119] Tang J., Yang J., Lu Q., Tang Q., Chen S., Jia G. (2022). The RNA N^6^-methyladenosine demethylase ALKBH9B modulates ABA responses in *Arabidopsis*. J. Integr. Plant Biol..

[120] Scutenaire J., Deragon J.M., Jean V., Benhamed M., Raynaud C., Favory J.J., Merret R., Bousquet-Antonelli C. (2018). The YTH domain protein ECT2 is an m^6^A reader required for normal trichome branching in *Arabidopsis*. Plant Cell.

[121] Li D., Zhang H., Hong Y., Huang L., Li X., Zhang Y., Ouyang Z., Song F. (2012). Genome-wide identification, biochemical characterization, and expression analyses of the YTH domain containing RNA-binding protein family in *Arabidopsis* and Rice. Plant Mol. Biol. Rep..

[122] Munns R., James R.A., Xu B., Athman A., Conn S.J., Jordans C., Byrt C.S., Hare R.A., Tyerman S.D., Tester M. (2012). Wheat grain yield on saline soils is improved by an ancestral Na^+^ transporter gene. Nat. Biotechnol..

[123] Venkataraman G., Shabala S., Véry A.A., Hariharan G.N., Somasundaram S., Pulipati S., Sellamuthu G., Harikrishnan M., Kumari K., Shabala L. (2021). To exclude or to accumulate? Revealing the role of the sodium HKT1;5 transporter in plant adaptive responses to varying soil salinity. Plant Physiol. Biochem..

[124] Olías R., Eljakaoui Z., Li J., De Morales P.A., Marín-Manzano M.C., Pardo J.M., Belver A. (2009). The plasma membrane Na^+^/H^+^ antiporter SOS1 is essential for salt tolerance in tomato and affects the partitioning of Na^+^ between plant organs. Plant Cell Environ..

[125] Egea I., Estrada Y., Faura C., Egea-Fernández J.M., Bolarin M.C., Flores F.B. (2023). Salt-tolerant alternative crops as sources of quality food to mitigate the negative impact of salinity on agricultural production. Front. Plant Sci..

[126] Wang Z., Hong Y., Li Y., Shi H., Yao J., Liu X., Wang F., Huang S., Zhu G., Zhu J.-K. (2021). Natural variations in SlSOS1 contribute to the loss of salt tolerance during tomato domestication. Plant Biotechnol. J..

[127] Wang X., Wang H., Liu S., Ferjani A., Li J., Yan J., Yang X., Qin F. (2016). Genetic variation in ZmVPP1 contributes to drought tolerance in maize seedlings. Nat. Genet..

[128] Matsukura S., Mizoi J., Yoshida T., Todaka D., Ito Y., Maruyama K., Shinozaki K., Yamaguchi-Shinozaki K. (2010). Comprehensive analysis of rice DREB2-type genes that encode transcription factors involved in the expression of abiotic stress-responsive genes. Mol. Genet. Genom..

[129] Zhang J., Zhang H., Srivastava A.K., Pan Y., Bai J., Fang J., Shi H., Zhu J.K. (2018). Knockdown of rice microRNA166 confers drought resistance by causing leaf rolling and altering stem xylem development. Plant Physiol..

[130] Lou D., Wang H., Yu D. (2018). The sucrose non-fermenting-1-related protein kinases SAPK1 and SAPK2 function collaboratively as positive regulators of salt stress tolerance in rice. BMC Plant Biol..

[131] Verma V., Kumar A., Partap M., Thakur M., Bhargava B. (2023). CRISPR-Cas: A robust technology for enhancing consumer-preferred commercial traits in crops. Front. Plant Sci..

[132] Abdallah N.A., Prakash C.S., McHughen A.G. (2015). Genome editing for crop improvement: Challenges and opportunities. GM Crops Food.

[133] Das D., Singha D.L., Paswan R.R., Chowdhury N., Sharma M., Reddy P.S., Chikkaputtaiah C. (2022). Recent advancements in CRISPR/Cas technology for accelerated crop improvement. Planta.

[134] Nguyen H.-C., Lin K.-H., Ho S.-L., Chiang C.-M., Yang C.-M. (2018). Enhancing the abiotic stress tolerance of plants: From chemical treatment to biotechnological approaches. Physiol. Plant.

[135] Gurmani A.R., Bano A., Ullah N., Khan H., Jahangir M.M., Flowers T.J. (2013). Exogenous abscisic acid (ABA) and silicon (Si) promote salinity tolerance by reducing sodium (Na^+^) transport and bypass flow in rice (‘*Oryza sativa*’ indica). Aust. J. Crop Sci..

[136] Sripinyowanich S., Klomsakul P., Boonburapong B., Bangyeekhun T., Asami T., Gu H., Buaboocha T., Chadchawan S. (2013). Exogenous ABA induces salt tolerance in indica rice (*Oryza sativa* L.): The role of *OsP5CS1* and *OsP5CR* gene expression during salt stress. Environ. Exp. Bot..

[137] Bulgakov V.P., Wu H.-C., Jinn T.-L. (2019). Coordination of ABA and chaperone signaling in plant stress responses. Trends Plant Sci..

[138] Liu X.-L., Zhang H., Jin Y.-Y., Wang M.-M., Yang H.-Y., Ma H.-Y., Jiang C.-J., Liang Z.-W. (2019). Abscisic acid primes rice seedlings for enhanced tolerance to alkaline stress by upregulating antioxidant defense and stress tolerance-related genes. Plant Soil.

[139] Sako K., Nguyen H.M., Seki M. (2021). Advances in chemical priming to enhance abiotic stress tolerance in plants. Plant Cell Physiol..

[140] Yuan L., Zheng Y., Nie L., Zhang L., Wu Y., Zhu S., Hou J., Shan G., Liu T., Chen G. (2021). Transcriptional profiling reveals changes in gene regulation and signaling transduction pathways during temperature stress in wucai (*Brassica campestris* L.). BMC Genom..

[141] Gour P., Kansal S., Agarwal P., Mishra B.S., Sharma D., Mathur S., Raghuvanshi S. (2022). Variety-specific transcript accumulation during reproductive stage in drought-stressed rice. Physiol. Plant.

[142] Liu Q., Wang F., Shuai Y., Huang L., Zhang X. (2022). Integrated analysis of single-molecule real-time sequencing and next-generation sequencing eveals insights into drought tolerance mechanism of *Lolium multiflorum*. Int. J. Mol. Sci..

[143] Mofatto L.S., Carneiro F.D.A., Vieira N.G., Duarte K.E., Vidal R.O., Alekcevetch J.C., Cotta M.G., Verdeil J.-L., Fabienne L.-M., Lartaud M. (2016). Identification of candidate genes for drought tolerance in coffee by high-throughput sequencing in the shoot apex of different *Coffea arabica* cultivars. BMC Plant Biol..

[144] Badhan S., Kole P., Ball A., Mantri N. (2018). RNA sequencing of leaf tissues from two contrasting chickpea genotypes reveals mechanisms for drought tolerance. Plant Physiol. Biochem..

